# Changes in NT-proBNP Levels According to SGLT2 Inhibitor Use in Patients Hospitalized for Acute Heart Failure Decompensation: A Prospective Cohort Study

**DOI:** 10.3390/life16040621

**Published:** 2026-04-08

**Authors:** Petros N. Fountoulakis, Panagiotis Theofilis, Evangelos Oikonomou, Gerasimos Siasos, Zoi Pallantza, Martha Bounta, Paschalis Karakasis, Panayotis K. Vlachakis, Konstantinos Tsioufis, Dimitris Tousoulis

**Affiliations:** 1 First Department of Cardiology, “Hippokration” General Hospital, University of Athens Medical School, 11527 Athens, Greece; pfountou@med.uoa.gr (P.N.F.); panos.theofilis@hotmail.com (P.T.); zoipallantza@gmail.com (Z.P.); marthabounta@gmail.com (M.B.); vlachakispanag@gmail.com (P.K.V.); ktsioufis@gmail.com (K.T.); 2 Third Department of Cardiology, Thoracic Diseases General Hospital “Sotiria”, University of Athens Medical School, 11527 Athens, Greece; boikono@gmail.com (E.O.); ger_sias@hotmail.com (G.S.); 3 Second Department of Cardiology, "Hippokration" General Hospital, Medical School, Aristotle University of Thessaloniki, 54642 Thessaloniki, Greece; pakar15@hotmail.com

**Keywords:** heart failure, SGLT2 inhibitor, natriuretic peptide

## Abstract

**Introduction:** Sodium-glucose cotransporter-2 inhibitors (SGLT2is) have proven beneficial in chronic heart failure (HF) across a wide range of left ventricular ejection fractions (LVEFs). Emerging data suggests that these benefits may extend to acute HF decompensation through enhanced decongestion. **Purpose:** To investigate changes in N-terminal pro-B-type natriuretic peptide (NT-proBNP) levels according to SGLT2i use among patients hospitalized for acute HF decompensation. **Methods:** In this prospective cohort study, consecutive patients hospitalized for HF decompensation were enrolled. Demographics, comorbidities, and cardiovascular risk factors were recorded. Participants were classified into three groups: Group 1—No SGLT2i use or discontinuation; Group 2—Prior SGLT2i use and continuation; Group 3—SGLT2i-naïve with initiation during hospitalization. NT-proBNP was measured on admission and discharge. **Results:** A total of 159 patients (median age 79 years, 64.8% male) were included. Group 1 patients exhibited negligible changes in NT-proBNP, whereas those continuing or newly initiating SGLT2i demonstrated significant reductions (absolute change: 506 [8792] pg/mL vs. −5610 [9461] pg/mL vs. −3602 [4409] pg/mL, *p* = 0.001, percentage change: −2.1 [63.4]% vs. −30.3 [39.0]% vs. −38.3 [41.5]%, *p* = 0.001). Multivariable regression confirmed that SGLT2i continuation or initiation independently predicted greater NT-proBNP reduction. **Conclusions:** NT-proBNP levels were significantly reduced among patients with decompensated HF treated with SGLT2i, with the greatest reduction in treatment-naïve patients. These findings highlight the potential role of SGLT2i even during acute HF hospitalization.

## 1. Introduction

Heart failure (HF) affects approximately 1–2% of the global population and poses major clinical and socioeconomic challenges [1]. With an aging population and increasing prevalence of cardiovascular comorbidities, HF hospitalizations are projected to rise by nearly 50% over the next 25 years [2]. HF results from structural and/or functional cardiac abnormalities leading to impaired ventricular filling or ejection [3]. According to European Society of Cardiology (ESC) guidelines, HF is classified by left ventricular ejection fraction (LVEF) into HF with reduced (HFrEF), mildly reduced (HFmrEF), or preserved ejection fraction (HFpEF) [4]. HF is also categorized by setting in chronic heart failure (CHF), the most common type, and acute heart failure (AHF) with an estimated prevalence between 1 and 12% of the general population [5,6]. AHF refers to an entity with rapid clinical manifestation on the background of a pre-existing cardiomyopathy whose underlying mechanisms are not totally elucidated [7]. Acute HF may usually be the result of acute myocardial infarction, hypertensive crisis, or chronic HF dysregulation [8]. AHF mostly affects male patients older than 65 years old and is often associated with poor prognosis, increased mortality ranging between 10% and 30% one-year post-discharge as well as higher risk of hospital readmissions approaching almost 30% during a 2–3 month follow-up period [7,9,10].

Despite therapeutic advances, HF continues to carry high morbidity and mortality [11]. Current treatment strategies comprise lifestyle modification and pharmacologic and device-based therapies [12]. Guideline-directed medical therapy includes angiotensin-converting enzyme inhibitors (ACEIs), angiotensin receptor blockers (ARBs) or angiotensin receptor–neprilysin inhibitors (ARNIs), β-blockers, mineralocorticoid receptor antagonists (MRAs), diuretics for congestion, and more recently, sodium-glucose cotransporter-2 inhibitors (SGLT2is) [13].

N-terminal pro-B-type natriuretic peptide (NT-proBNP) is a well-established biomarker of myocardial wall stress, congestion, and prognosis in HF [14,15]. NT-proBNP is produced by cardiomyocytes in response to stimulation or myocardial stretch and is responsible for the regulation of blood pressure and volume as well as sodium balance [16]. Elevated NT-proBNP levels correlate with worse outcomes and are used for diagnosis, risk stratification, and therapy monitoring [17]. SGLT2i trials in chronic HF populations have shown significant NT-proBNP reduction after 12 weeks of treatment [18,19]. However, data regarding NT-proBNP responses to SGLT2i initiation or continuation during acute HF hospitalization remain limited. This study aimed to evaluate the pattern of NT-proBNP changes in patients hospitalized for acute HF decompensation according to SGLT2i utilization.

## 2. Methods

### 2.1. Study Population

In this prospective cohort study, we enrolled consecutive patients hospitalized for HF decompensation at the 1st Department of Cardiology, “Hippokration” General Hospital of Athens, between May 2023 and February 2024. We included adult patients aged ≥18 years, with a primary diagnosis of acute HF decompensation, eligible for SGLT2i therapy, who had baseline and discharge NT-proBNP assessments. We excluded patients with contraindications to SGLT-2i (severe renal impairment with eGFR <20 mL/min/1.73 m^2^, history of diabetic ketoacidosis, type 1 DM), missing baseline or discharge NT-proBNP assays, and pregnant women. Patients with an expected survival of <3 months were also excluded to avoid inclusion of individuals with severe non-cardiac comorbidities or terminal illnesses that could substantially influence biomarker levels and clinical management during hospitalization. Patients were followed throughout the index hospitalization, and the observation period extended from hospital admission until discharge, when the second NT-proBNP measurement was obtained.

Prior medical history and cardiovascular risk factors were recorded. The patterns in SGLT2i use were noted, and patients were classified into three groups:

• Group 1 (No SGLT2i use/discontinuation).
• Group 2 (Prior SGLT2i use and continuation).
• Group 3 (SGLT2i-naïve and initiation).

Treatment allocation was not randomized, and patterns of SGLT2i use (continuation, discontinuation, or in-hospital initiation) reflected routine clinical decision making by the treating physicians in this prospective observational cohort. In patients categorized in Group 1, SGLT2is were either not prescribed prior to admission or were discontinued at the time of hospitalization according to clinical judgment.

This study complied with the Declaration of Helsinki, and all participants provided written informed consent. This study was approved by the Institutional Review Board of the Hippokration Hospital (protocol code: 172, date of approval: 15 February 2023).

### 2.2. Laboratory Investigations

We assessed the following laboratory biomarkers:

1. Creatinine with biochemical photometry ‘Abbott Alinity’ analyzer.
2. Renal function with estimated glomerular filtration rate (eGFR) calculated with Cockcroft–Gault equation.
3. High-Sensitivity Troponine I (hsTnI) with chemiluminescent microparticle immunoassay by ‘Abbott Alinity’ analyzer.
4. N-terminal prohormone of brain natriuretic peptide (NT-proBNP) with electrochemiluminescence immunoassay by ‘Roche’s Elecsys’ analyzer.
5. High-Sensitivity CRP (hsCRP) with ‘Abbott Alinity’ chemistry analyzer.

### 2.3. Echocardiography

All subjects underwent standard transthoracic echocardiography using the GE Vivid™ E90 ultrasound system (manufactured by GE HealthCare, Chicago, IL, USA). The examinations were performed by a single experienced operator in a dimly lit room to optimize image acquisition. The acquired images were analyzed using the EchoPAC Clinical Workstation.

Left ventricular ejection fraction (LVEF) was assessed using the biplane Simpson’s method, which involves tracing the endocardial border in both the apical four-chamber and two-chamber views at end-diastole and end-systole [20]. This technique calculates LVEF by dividing the difference between end-diastolic and end-systolic volumes by the end-diastolic volume, providing a more accurate assessment of global left ventricular systolic function compared to linear methods.

### 2.4. Statistical Analysis

Continuous variables were assessed for normality using the Kolmogorov–Smirnov test and visual inspection of probability–probability (P-P) plots. Depending on the distribution, data were expressed as mean ± standard deviation (SD) for normally distributed variables or as median with interquartile range (IQR: 25th, 75th percentile) for non-normally distributed variables. For normally distributed data one-way analysis of variance (ANOVA) was performed to examine for intergroup differences, as appropriate. The Kruskal–Wallis ANOVA was used for variables not following a normal distribution. Post hoc analysis was performed using the Bonferroni correction. Categorical variables were analyzed using contingency tables and χ^2^ tests. To investigate the association between percentage change in NT-proBNP and other study variables, including patterns of SGLT2i utilization, a multivariable linear regression analysis was performed. Patterns of SGLT2i utilization were entered into the regression model using dummy-variable coding, with the “No SGLT2i use/discontinuation” group serving as the reference category.

All statistical tests were two-sided, with significance set at *p* < 0.05. Statistical analyses were conducted using IBM SPSS software (IBM Corp. Released 2019. IBM SPSS Statistics for Windows, Version 26.0, Armonk, NY, USA).

## 3. Results

### 3.1. Characteristics of the Study Population

**Figure 1:**
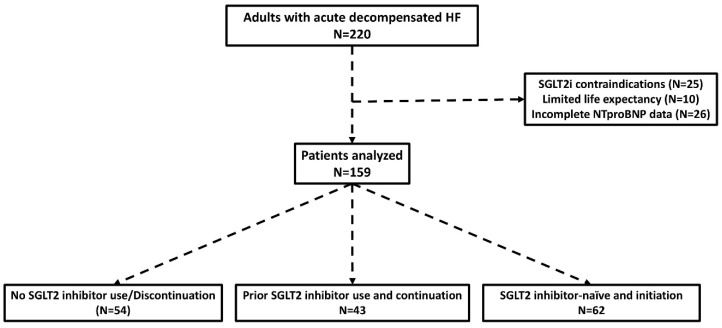
Study flow diagram.

A total of 220 patients with acute HF decompensation were initially considered (Figure 1). After application of the exclusion criteria, 159 patients were included and analyzed (median age: 79 years old, male sex: 64.8%). The observation period corresponded to the hospitalization period (median duration of hospitalization: 6 days). In this population, 67.9% of the patients were not receiving an SGLT2i on admission. The baseline characteristics of the study population according to SGLT2i use are displayed in Table 1. There were no major differences in age, sex distribution, history of diabetes mellitus, atrial fibrillation, or renal function. Group 2 patients had a lower prevalence of arterial hypertension compared to Group 3 (36.6% vs. 52.6%, *p* < 0.05). Patients of Group 1 had the highest prevalence of HFrEF and the lowest prevalence of HFpEF.

### 3.2. Changes in NT-proBNP According to SGLT2i Use

**Figure 2:**
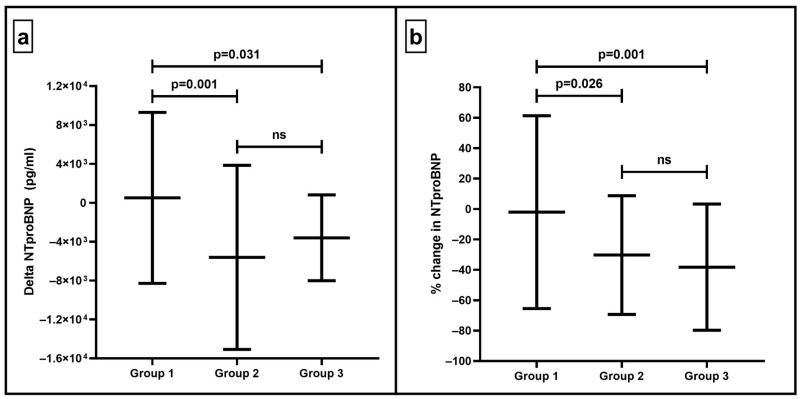
Changes in NT-proBNP concentrations of patients hospitalized for HF decompensation in response to different patterns of SGLT2i utilization. Group 1 (no SGLT2i use/discontinuation), Group 2 (prior SGLT2i use and continuation), and Group 3 (SGLT2i-naïve and initiation). (**a**) Absolute change in NTproBNP. (**b**) Percenage change in NTproBNP.

Moving to the changes in NT-proBNP (Figure 2), we observed that patients who did not use SGLT2is and did not initiate them during hospitalization had negligible changes in NT-proBNP, either as an absolute change (Group 1: 506 (8792) pg/mL vs. Group 2: −5610 (9461) pg/mL vs. Group 3: −3602 (4409) pg/mL, *p* = 0.001) or as percentage change (Group 1: −2.1 (63.4) % vs. Group 2: −30.3 (39.0) % vs. Group 3: -38.3 (41.5) %, *p* = 0.001), compared to the rest of the study population who were already taking SGLT2is or initiated them during hospitalization. Interestingly, according to a multivariable regression analysis, the continuation or initiation of SGLT2i (compared to no use/discontinuation) was significantly associated with the greatest percentage change in NT-proBNP (Table 2).

## 4. Discussion

In this study of patients hospitalized for acute heart failure decompensation, we observed that patients who stopped treatment with SGLT2i and/or did not initiate them during hospitalization had insignificant changes in NT-proBNP levels. The most favorable results were observed in the HF population with prior SGLT2i use and continuation as well as in patients who had never received this medication and initiated during hospitalization. It is noteworthy that treatment-naïve HF patients revealed the most significant reduction in NT-proBNP concentrations when compared to prior SGLT2i use and continuation.

SGLT2is exert cardioprotection through complementary hemodynamic and cellular pathways that extend beyond glucose lowering. Proximally, they reduce renal glucose-sodium reabsorption, restore tubuloglomerular feedback, and induce natriuresis/osmotic diuresis, which lowers preload/afterload and contributes to sustained blood-pressure reductions alongside improvements in arterial stiffness, endothelial function, sympathetic tone, body weight, and uric acid handling [21]. At the tissue level, SGLT2is remodel adverse myocardial biology: they dampen inflammatory signaling with consistent decreases in circulating IL-6, CRP, TNF-α, and MCP-1 in experimental models, supporting a systemic anti-inflammatory effect [22]. They also mitigate cardiac fibrosis by modulating oxidative stress and mitochondrial function and by restraining canonical profibrotic cascades (e.g., TGF-β/Smad, STAT3, mTOR), while promoting autophagy and favorable energetic shifts (greater fatty-acid/ketone utilization). These actions appear in diabetic and non-diabetic settings and are increasingly corroborated by imaging and biomarker data [23]. In parallel, SGLT2is reduce epicardial adipose tissue, an inflammatory, pro-fibrotic depot contiguous with the myocardium, offering a plausible paracrine route for additional benefit [24]. Collectively, these mechanisms (hemodynamic unloading, anti-inflammation/antioxidation, antifibrotic remodeling, improved bioenergetics, and vascular/autonomic effects) provide a coherent rationale for the rapid NT-proBNP declines and HF outcome improvements observed with SGLT2is across the LVEF spectrum [25].

SGLT2is have been tried in patients with acute decompensated HF. In the double-blind EMPULSE trial (n = 530), patients hospitalized for acute de novo or decompensated chronic HF were randomized when clinically stable (median of 3 days after admission) to empagliflozin 10 mg or placebo for 90 days [26]. The hierarchical primary endpoint (all-cause death, number/time to HF events, and ≥5-point KCCQ-TSS improvement) was met (stratified win ratio 1.36, 95% CI 1.09–1.68, *p* = 0.0054). Empagliflozin also yielded greater early NT-proBNP reduction (day-30 AUC adjusted geometric mean ratio 0.90, 95% CI 0.82–0.98) with a favorable safety profile. Benefits were consistent across de novo vs. decompensated HF, across the EF spectrum, and irrespective of diabetes. A prespecified analysis of EMPULSE examined a spectrum of decongestion markers, body-weight loss, weight loss indexed to loop-diuretic dose, hemoconcentration (hematocrit), clinical congestion score, and NT-proBNP AUC at 15, 30, and 90 days [27]. Empagliflozin produced greater weight loss than placebo at each time point (adjusted mean differences −1.97 kg at day 15, −1.74 kg at day 30, and −1.53 kg at day 90, all *p* < 0.05) and, importantly, greater weight loss per diuretic dose (−2.31, −2.79, and −3.18 kg/40 mg furosemide-equivalent at days 15, 30, and 90), indicating enhanced diuretic efficiency rather than simply higher diuretic exposure. Hematocrit rose more with empagliflozin at all time points (adjusted mean differences ~1.6-1.9%), consistent with intravascular decongestion, while clinical congestion scores also improved more at day 15. NT-proBNP trajectories favored empagliflozin early (adjusted geometric mean ratio for AUC vs. placebo 0.92 at day 15 and 0.90 at day 30) with a borderline effect by day 90 (0.89, *p* = 0.056). Notably, patients achieving greater weight loss or larger hematocrit increases by day 15/30 had a higher probability of clinical benefit at day 90 on the hierarchical composite, linking decongestion magnitude to subsequent clinical success. Together, these data complement the primary EMPULSE findings by showing early, effective, and sustained decongestion that plausibly mediates the short-term clinical advantages of in-hospital SGLT2i initiation.

Our study shows that continuation or in-hospital initiation of SGLT2i during acute HF decompensation is associated with a significant reduction in NT-proBNP, compared with non-use or discontinuation. Large randomized trials (e.g., DAPA-HF, DECLARE-TIMI 58, EMPA-REG OUTCOME) consistently support favorable effects of SGLT2i on HF biomarkers and on clinical outcomes, including reduced mortality and HF hospitalization, across the LVEF spectrum [28]. In a meta-analysis by Chen et al., SGLT2i therapy yielded >20% reductions in NT-proBNP and modest improvements in 6 min walk distance and left ventricular systolic function (~3%) [29]. Our findings also align with Fukuta et al., who reported improved NT-proBNP and exercise capacity in HFpEF with SGLT2i therapy [30]. Apart from that, another recent publication by Santos Guzmán et al. focused on individuals with HFpEF under SGLT2i treatment, which showed lower NT-proBNP together with reductions in cardiovascular mortality and HF hospitalizations in treated individuals [31]. Conversely, Packer et al. demonstrated that withdrawing SGLT2i for approximately 4 weeks led to rapid increases in NT-proBNP and adverse events, alongside reversal of prior benefits on blood pressure, body weight, renal indices, and metabolic markers [32].

An additional observation in our cohort was that SGLT2i-naïve patients experienced greater NT-proBNP reductions than prior, continuous users. This is biologically plausible: early hemodynamic unloading, anti-inflammatory effects, and metabolic shifts may confer the largest incremental gains in previously untreated individuals. Supporting this, Laborante et al. found that immediate in-hospital initiation of SGLT2i reduced NT-proBNP and HF rehospitalization risk [33]. The EMMY trial similarly showed that empagliflozin in the acute coronary syndrome setting improved NT-proBNP, LVEF, and filling pressures [34]. Furthermore, the addition of dapagliflozin to standard HF therapy in T2DM improved LV structure and lowered NT-proBNP over 5 years [35].

Although SGLT2is possess diuretic-like properties, this natriuretic/osmotic diuretic effect is transient and unlikely to fully explain sustained biomarker changes. Multiple studies suggest that SGLT2is enhance decongestion acutely while also improving vascular function, autonomic balance, myocardial energetics, and antifibrotic signaling, thereby supporting ongoing NT-proBNP improvement independent of persistent diuresis [36]. Attenuation of the diuretic signal may reflect adaptive mechanisms such as vasopressin-mediated water conservation and reduced natriuresis over time [37]. In RECEDE-CHF, empagliflozin combined with loop diuretics produced a significant but short-lived natriuretic response that diminished by approximately 3 months [38].

This study has several limitations. First, it was a non-randomized, single-center cohort in which treatment groups (continuation, initiation, or discontinuation/absence of SGLT2i) were determined by routine clinical practice rather than random allocation. As such, confounding by indication and local practice patterns cannot be excluded. The sample size was modest, with attrition from initial screening to final analysis, which reduces statistical power and may introduce selection bias. NT-proBNP was measured only at admission and at discharge, providing a short observation period without post-discharge follow-up. Consequently, we cannot determine whether the observed biomarker changes translate into differences in clinical outcomes such as rehospitalization or mortality. In addition, detailed functional status parameters such as NYHA class were not recorded in the study database, while LVEF was not systematically reassessed at discharge, limiting the ability to evaluate short-term changes in cardiac function during hospitalization. Heart-failure phenotypes (HFrEF, HFmrEF, HFpEF) were heterogeneous and imbalanced across groups, creating potential confounding despite adjustment. In-hospital care, including diuretic strategies, titration of other guideline-directed medical therapies, fluid balance, and discharge timing, was not standardized and may have influenced NT-proBNP trajectories. Generalizability is also limited, as the cohort was derived from an older inpatient population at a single tertiary center, which may not reflect different groups such as younger patients, outpatients, or other health-care settings. Finally, we did not assess adherence after discharge, renal or hemodynamic endpoints. These factors support interpreting the findings as hypothesis-generating and reveal the need for randomized, multi-center studies with standardized co-therapies and longer follow-up.

## 5. Conclusions

In this Greek cohort of patients hospitalized for acute decompensated HF, absence or discontinuation of SGLT2i was associated with minimal NT-proBNP change, whereas continuation or in-hospital initiation correlated with substantial NT-proBNP reductions, most pronounced in treatment-naïve patients. These findings support early initiation and sustained use of SGLT2i, alongside other pillars of guideline-directed HF therapy, to enhance decongestion and improve biomarker profiles in the acute setting. Confirmation in randomized, multicenter studies with longer follow-up is warranted to determine whether these biomarker improvements translate into durable reductions in rehospitalization and mortality across HF phenotypes.

## Figures and Tables

**Table 1:** Characteristics of the study population according to the patterns in SGLT2i use.

Parameter	Group 1 (N = 54)	Group 2 (N = 43)	Group 3 (N = 62)	*p*
Age, years	75.4 (15.1)	74.8 (11.7)	78.7 (9.0)	0.20
Male sex, %	55.6	69.0	72.1	0.15
Hypertension, %	57.4	36.6	72.6 ^†^	0.001
Diabetes mellitus, %	27.8	50.0	43.5	0.07
Atrial fibrillation, %	63.0	71.4	71.0	0.58
LVEF, %	43 (11)	32 (12)	39 (11)	<0.001
eGFR (admission), mL/min/1.73 m^2^	42 (28, 70)	38 (30, 51)	52 (36, 65)	0.09
hsTnI (admission), pg/mL	27.6 (13.1, 52.8)	42.2 (14.0, 128.8)	24.6 (12.3, 49.3)	0.33
hsCRP (admission), mg/L	9.5 (4.5, 44.0)	7.8 (2.3, 16.7)	8.1 (3.8, 23.4)	0.22
HF category				
HFrEF, %	31.5	71.4 *	46.8	0.002
HFmrEF, %	20.4	9.5	24.2	
HFpEF, %	48.1	19.0 *	29.0 ^†^	

eGFR: estimated glomerular filtration rate, hsTnI: high-sensitivity Troponin I, hsCRP: high-sensitivity C-reactive protein, HF: HF, HFrEF: HF with reduced ejection fraction, HFmrEF: HF with mildly reduced ejection fraction, HFpEF: HF with preserved ejection fraction. *: denotes statistically significant difference (*p* < 0.05) compared to Group 1. ^†^: denotes statistically significant difference (*p* < 0.05) compared to Group 2.

**Table 2:** Regression analysis of variables associated with percentage change in NT-proBNP.

Variable	β Coefficient	95% CI	*p*
Age	0.62	−0.19, 1.42	0.13
Male sex	15.00	−5.31, 35.30	0.15
Hypertension	6.59	−12.69, 25.88	0.50
T2DM	−8.32	−27.23, 10.60	0.39
AF	−8.88	−28.93, 11.18	0.38
eGFR (admission)	−0.21	−0.67, 0.25	0.37
hsTnI (admission)	−0.01	−0.03, 0.01	0.24
hsCRP (admission)	−0.02	−0.38, 0.34	0.91
HFpEF (Ref)			
HFmrEF	−3.29	−29.69, 23.10	0.81
HFrEF	−0.08	−21.68, 21.53	0.99
No SGLT2i use/discontinuation (Ref)			
Prior SGLT2i use and continuation	−28.96	−54.39, −3.52	0.026
SGLT2i-naïve and initiation	−39.40	−61.41, −17.40	0.001
Overall R^2^: 0.156, *p*-value: 0.013			

## Data Availability

The data supporting the findings of this manuscriopt is available upon reasonable request to the correspoding author.

## References

[1] Walia R.S., Mankoff R. (2023). Impact of Socioeconomic Status on Heart Failure. J. Community Hosp. Intern. Med. Perspect..

[2] Savarese G., Lund L.H. (2017). Global Public Health Burden of Heart Failure. Card. Fail. Rev..

[3] Bozkurt B., Coats A.J., Tsutsui H., Abdelhamid M., Adamopoulos S., Albert N., Anker S.D., Atherton J., Böhm M., Butler J. (2021). Universal Definition and Classification of Heart Failure: A Report of the Heart Failure Society of America, Heart Failure Association of the European Society of Cardiology, Japanese Heart Failure Society and Writing Committee of the Universal Definition of Heart Failure. J. Card. Fail..

[4] McDonagh T.A., Metra M., Adamo M., Gardner R.S., Baumbach A., Böhm M., Burri H., Butler J., Čelutkienė J., Chioncel O. (2021). 2021 ESC Guidelines for the diagnosis and treatment of acute and chronic heart failure: Developed by the Task Force for the diagnosis and treatment of acute and chronic heart failure of the European Society of Cardiology (ESC) With the special contribution of the Heart Failure Association (HFA) of the ESC. Eur. Heart J..

[5] Kurmani S., Squire I. (2017). Acute Heart Failure: Definition, Classification and Epidemiology. Curr. Heart Fail. Rep..

[6] Searle J., Frick J., Möckel M. (2016). Acute heart failure facts and numbers: Acute heart failure populations. ESC Heart Fail..

[7] Arrigo M., Jessup M., Mullens W., Reza N., Shah A.M., Sliwa K., Mebazaa A. (2020). Acute heart failure. Nat. Rev. Dis. Primers.

[8] Heidenreich P.A., Bozkurt B., Aguilar D., Allen L.A., Byun J.J., Colvin M.M., Deswal A., Drazner M.H., Dunlay S.M., Evers L.R. (2022). 2022 AHA/ACC/HFSA Guideline for the Management of Heart Failure: A Report of the American College of Cardiology/American Heart Association Joint Committee on Clinical Practice Guidelines. Circulation.

[9] Farmakis D., Filippatos G., Tubaro M., Vranckx P., Bonnefoy-Cudraz E., Price S., Vrints C., Tubaro M., Vranckx P., Price S., Vrints C., Bonnefoy E. (2021). Acute heart failure: Epidemiology, classification, and pathophysiology. The ESC Textbook of Intensive and Acute Cardiovascular Care.

[10] Montalto M., D’Ignazio F., Camilli S., Di Francesco S., Fedele M., Landi F., Gallo A. (2025). Heart Failure in Older Patients: An Update. J. Clin. Med..

[11] Savarese G., Becher P.M., Lund L.H., Seferovic P., Rosano G.M.C., Coats A.J.S. (2023). Global burden of heart failure: A comprehensive and updated review of epidemiology. Cardiovasc. Res..

[12] Palaparthi E.C., K P., Ignasimuthu A., N G., Sade N., Bade N., Jakka B.K., Gogineni K.K., Dunde A., Medabala T. (2025). Impact of Lifestyle Modifications Along With Pharmacological Treatment of Heart Failure: A Narrative Review. Cureus.

[13] McDonagh T.A., Metra M., Adamo M., Gardner R.S., Baumbach A., Böhm M., Burri H., Butler J., Čelutkienė J., Chioncel O. (2023). 2023 Focused Update of the 2021 ESC Guidelines for the diagnosis and treatment of acute and chronic heart failure: Developed by the task force for the diagnosis and treatment of acute and chronic heart failure of the European Society of Cardiology (ESC) With the special contribution of the Heart Failure Association (HFA) of the ESC. Eur. Heart J..

[14] Tsutsui H., Albert N.M., Coats A.J.S., Anker S.D., Bayes-Genis A., Butler J., Chioncel O., Defilippi C.R., Drazner M.H., Felker G.M. (2023). Natriuretic Peptides: Role in the Diagnosis and Management of Heart Failure: A Scientific Statement From the Heart Failure Association of the European Society of Cardiology, Heart Failure Society of America and Japanese Heart Failure Society. J. Card. Fail..

[15] Yilmaz Oztekin G.M., Genc A., Cagirci G., Arslan S. (2022). Prognostic value of the combination of uric acid and NT-proBNP in patients with chronic heart failure. Hellenic J. Cardiol..

[16] Novack M.L., Zubair M. (2025). Natriuretic Peptide B Type Test. StatPearls.

[17] Daubert M.A., Adams K., Yow E., Barnhart H.X., Douglas P.S., Rimmer S., Norris C., Cooper L., Leifer E., Desvigne-Nickens P. (2019). NT-proBNP Goal Achievement Is Associated With Significant Reverse Remodeling and Improved Clinical Outcomes in HFrEF. JACC Heart Fail..

[18] McMurray J.J.V., Solomon S.D., Inzucchi S.E., Køber L., Kosiborod M.N., Martinez F.A., Ponikowski P., Sabatine M.S., Anand I.S., Bělohlávek J. (2019). Dapagliflozin in Patients with Heart Failure and Reduced Ejection Fraction. N. Engl. J. Med..

[19] Nassif M.E., Windsor S.L., Tang F., Khariton Y., Husain M., Inzucchi S.E., McGuire D.K., Pitt B., Scirica B.M., Austin B. (2019). Dapagliflozin Effects on Biomarkers, Symptoms, and Functional Status in Patients With Heart Failure With Reduced Ejection Fraction: The DEFINE-HF Trial. Circulation.

[20] Mitchell C., Rahko P.S., Blauwet L.A., Canaday B., Finstuen J.A., Foster M.C., Horton K., Ogunyankin K.O., Palma R.A., Velazquez E.J. (2019). Guidelines for Performing a Comprehensive Transthoracic Echocardiographic Examination in Adults: Recommendations from the American Society of Echocardiography. J. Am. Soc. Echocardiogr..

[21] Katsimardou A., Theofilis P., Vordoni A., Doumas M., Kalaitzidis R.G. (2024). The Effects of SGLT2 Inhibitors on Blood Pressure and Other Cardiometabolic Risk Factors. Int. J. Mol. Sci..

[22] Theofilis P., Sagris M., Oikonomou E., Antonopoulos A.S., Siasos G., Tsioufis K., Tousoulis D. (2022). The impact of SGLT2 inhibitors on inflammation: A systematic review and meta-analysis of studies in rodents. Int. Immunopharmacol..

[23] Karakasis P., Theofilis P., Vlachakis P.K., Apostolos A., Milaras N., Ktenopoulos N., Grigoriou K., Klisic A., Karagiannidis E., Fyntanidou B. (2025). SGLT2 inhibitors and cardiac fibrosis: A comprehensive review. Curr. Probl. Cardiol..

[24] Theofilis P., Oikonomou E., Vlachakis P.K., Karakasis P., Dimitriadis K., Sagris M., Pamporis K., Drakopoulou M., Siasos G., Tsioufis K. (2025). Sodium-Glucose Cotransporter 2 Inhibitors and Changes in Epicardial Adipose Tissue: A Systematic Literature Review And Meta-Analysis. Curr. Vasc. Pharmacol..

[25] Piperis C., Marathonitis A., Anastasiou A., Theofilis P., Mourouzis K., Giannakodimos A., Tryfou E., Oikonomou E., Siasos G., Tousoulis D. (2024). Multifaceted Impact of SGLT2 Inhibitors in Heart Failure Patients: Exploring Diverse Mechanisms of Action. Biomedicines.

[26] Voors A.A., Angermann C.E., Teerlink J.R., Collins S.P., Kosiborod M., Biegus J., Ferreira J.P., Nassif M.E., Psotka M.A., Tromp J. (2022). The SGLT2 inhibitor empagliflozin in patients hospitalized for acute heart failure: A multinational randomized trial. Nat. Med..

[27] Biegus J., Voors A.A., Collins S.P., Kosiborod M.N., Teerlink J.R., Angermann C.E., Tromp J., Ferreira J.P., Nassif M.E., Psotka M.A. (2023). Impact of empagliflozin on decongestion in acute heart failure: The EMPULSE trial. Eur. Heart J..

[28] Chen Y.R., Zhu F.Y., Zhou R. (2024). SGLT2 inhibitors for alleviating heart failure through non-hypoglycemic mechanisms. Front. Cardiovasc. Med..

[29] Chen J., Jiang C., Guo M., Zeng Y., Jiang Z., Zhang D., Tu M., Tan X., Yan P., Xu X. (2024). Effects of SGLT2 inhibitors on cardiac function and health status in chronic heart failure: A systematic review and meta-analysis. Cardiovasc. Diabetol..

[30] Fukuta H., Hagiwara H., Kamiya T. (2022). Sodium-glucose cotransporter 2 inhibitors in heart failure with preserved ejection fraction: A meta-analysis of randomized controlled trials. Int. J. Cardiol. Heart Vasc..

[31] Santos Guzmán M.V., Rivera D., Reyna Guerrero I.M., Fernandez Rodriguez A.L., Azcona R., Escobar Batista D., Montano Diaz G., García Zuluaga L.C., Santos Rosario V. (2025). Efficacy of Sodium-Glucose Cotransporter 2 (SGLT2) Inhibitors in Heart Failure With Preserved Ejection Fraction: A Systematic Review. Cureus.

[32] Packer M., Butler J., Zeller C., Pocock S.J., Brueckmann M., Ferreira J.P., Filippatos G., Usman M.S., Zannad F., Anker S.D. (2023). Blinded Withdrawal of Long-Term Randomized Treatment With Empagliflozin or Placebo in Patients With Heart Failure. Circulation.

[33] Laborante R., Paglianiti D.A., Bianchini E., Galli M., Borovac J.A., Savarese G., Patti G., D’Amario D. (2025). Safety and efficacy of early initiation of sodium-glucose co-transporter inhibitors 2 in patients hospitalized for acute heart failure: A meta-analysis of randomized controlled trials. Eur. J. Intern. Med..

[34] Kotit S. (2023). EMMY: The continued expansion of clinical applications of SGLT2 inhibitors. Glob. Cardiol. Sci. Pract..

[35] Yu P.L., Yu Y., Li S., Mu B.C., Nan M.H., Pang M. (2024). Dapagliflozin in heart failure and type 2 diabetes: Efficacy, cardiac and renal effects, safety. World J. Diabetes.

[36] Munteanu M.A., Swarnkar S., Popescu R.I., Lungu A., Ciobotaru L., Nicolae C., Tufanoiu E., Nanea I.T. (2023). SGLT2 Inhibitor: An Emerging Pillar in Heart Failure Therapeutics?. Maedica.

[37] Tang J., Ye L., Yan Q., Zhang X., Wang L. (2022). Effects of Sodium-Glucose Cotransporter 2 Inhibitors on Water and Sodium Metabolism. Front. Pharmacol..

[38] McLean P., Bennett J., “Trey” Woods E., Chandrasekhar S., Newman N., Mohammad Y., Khawaja M., Rizwan A., Siddiqui R., Birnbaum Y. (2025). SGLT2 inhibitors across various patient populations in the era of precision medicine: The multidisciplinary team approach. NPJ Metab. Health Dis..

